# *In vitro* and *in vivo* degradation behavior of poly(trimethylene carbonate-co-d,l-lactic acid) copolymer

**DOI:** 10.1093/rb/rbx003

**Published:** 2017-07-07

**Authors:** Zhengyu Ma, Yi Wu, Jing Wang, Changsheng Liu

**Affiliations:** 1 Engineering Research Center for Biomedical Materials of Ministry of Education, School of Materials Science and Engineeering, East China University of Science and Technology, Shanghai 200237, People’s Republic of China; 2 The State Key Laboratory of Bioreactor Engineering, School of Materials Science and Engineering, East China University of Science and Technology, Shanghai 200237, People’s Republic of China; 3 Key Laboratory for Ultrafine Materials of Ministry of Education, School of Materials Science and Engineering, East China University of Science and Technology, Shanghai 200237, People’s Republic of China

**Keywords:** P(TMC-co-DLLA), copolymer, degradation, tissue repair

## Abstract

We present P(TMC-co-DLLA) copolymer with the molar ratio of TMC: DLLA = 15: 85 was used to systematic study of *in vivo* and *in vitro* degradation behaviors. Dense homogeneous copolymer specimens were prepared by compression molding method. The *in vitro* and *in vivo* degradation were, respectively, performed at simulative body condition and implanted into rat’s subcutaneous condition. Investigations were followed via physicochemical and histological analysis such as SEM, GPC, DSC, FTIR and H&E stain. The results demonstrate that copolymeric material can degrade in phosphate buffer solution (PBS) and in rat’s body, and the *in vivo* degradation rate is faster. Obvious decline of molecule weight and mass loss has been observed, which led to the attenuation of mechanical strength. Furthermore, apart from the hydrolysis, macrophagocytes took part in the phagocytosis *in vivo*, indicating that degradation rate could be regulated by the combinational mechanism. It is concluded that P(TMC-co-DLLA) copolymer is a promising candidate for tissue repair.

## Introduction

Synthetic biodegradable polymers have been studied proverbially and widely used for biomedical applications because of their excellent biodegradability, mechanical properties, minor cytotoxicity and immunogenicity [1–6]. However, most homopolymers commercially available cannot possess perfect integrated properties. For instance, PCL-TMC has more outstanding anticoagulant properties than PCL and PTMC [7]. As reported [8], PGS-co-PEG polymers showed better water uptake capacity and mechanical stability compared with PGS. Therefore copolymers composed of different blocks are likely to improve the combinational behaviors including biodegradation rate, mechanical properties and cell affinity behavior, etc [9–12].

PDLLA and PTMC are well known polymers that can be applied in intravitreal delivery systems or bone defect [13–15], mostly in the form of scaffolds and microspheres [16, 17]. Other than the ‘bulk degradation mode’ of PDLLA, PTMC has been shown to degrade by surface erosion approach [18, 19], avoiding the sharp attenuation of mechanical strength during fragmentation of the matrix. In addition, PDLLA cleaves by hydrolysis into acidulous lactic acid [20, 21], which has an adverse effect on surrounding tissue. It has been demonstrated that enzymes and reactive oxygen species secreted by phagocytic cells play an important role in the *in vivo* degradation of PTMC [22, 23], and it degrades without the formation of acidic compounds, which is an advantage as regenerative material. In view of the different degradation mechanism between PDLA and PTMC, copolymerization with variant of TMC and DLLA may be an effective strategy to regulate degradation behavior [19, 24–26]. According to another investigation of Feijen [27, 28], the TMC-co-DLLA copolymers degraded faster than the parent homopolymers and the higher the DLLA content was, the faster the degradation rate was. The biodegradable feature of flexible PTMC and its copolymers with *ε*-caprolactone or D, L-lactide has been investigated *in vitro* and *in vivo*, which is beneficial in tissue repairing [29].

In our previous work, we synthesized PTMC-PDLA copolymers randomly via ring-opening polymerization, thereinto P(TMC-co-DLLA) copolymers (15:85) was selected to be used as scaffolds for bone repairing. Herein we studied its *in vitro* degradation behaviors, including variations of surface morphology, water uptake, mass loss, and deterioration of mechanical properties. Moreover, the *in vivo* degradation and histocompatibility were inspected by subcutaneous implantation. The objective of this work was to provide more detailed information about the degradation features of P(TMC-co-DLLA) as tissue repairing material.

## Materials and methods

### Materials

D, L-lactide (DLLA) was purchased from J&K scientific and recrystallized in anhydrous toluene for three times. Trimethylene carbonate (TMC) was obtained from Jinan Daigang Biomaterial Co., Ltd and recrystallized in ethyl acetate. Dichloromethane (CH_2_Cl_2_) and anhydrous methanol (CH_3_OH) were purchased from Sinopharm Chemical Reagent Co., Ltd. Tetrahydrofuran (THF), pentaerythrite (C(CH_2_OH)_4_), and stannous octoate (Sn(Oct)_2_) were purchased from Shanghai Lingfeng Chemical Reagent Co., Ltd. Ethyl acetate (C_4_H_8_O_2_) was distilled from P_2_O_5_. Dimethylben (C_6_H_4_ (CH_3_)_2_) was distilled from sodium/benzophenone in an inert atmosphere to remove water before using. All other reagents were of analytical grade and used as received. Phosphate buffer solution (PBS, pH 7.4) was lab-made.

### Synthesis of P(TMC-co-DLLA) copolymer

P(TMC-co-DLLA) was synthesized by bulk melt polymerization according to the method reported [10]. Briefly, silanized *schlenk* tube was dried at 120°C and purged by argon for 3–5 times. Purified TMC and D, L-LA with the molar ratio of 15: 85 were mixed and put into *schlenk* tube. Stannous octoate (Sn(Oct)_2_: DLLA = 1: 5000, molar ratio) and pentaerythrite (initiator: monomer = 1: 402, molar ratio) were transferred into the *schlenk* tube, serving as catalyst and initiator, respectively. The polymerization tube was purged with argon and degassed for 30 min by vacuum to remove oxygen and trace water. Then tube was sealed under vacuum. The polymerization was conducted at 130°C for 72 h. After polymerization, the crude products were dissolved in methylene chloride solution, and precipitated from methanol. The obtained copolymer was thoroughly vacuum-dried at room temperature for 48 h.

### *In vitro* degradation

P(TMC-co-DLLA) copolymer specimens were fabricated by compression molding at 140°C under 5 MPa, and then cut into strips with dimensions of 2× 2 × 4 mm.

The degradation study was performed in a phosphate buffer saline solution (PBS) with pH = 7.4. Firstly, the weighed specimen (*W_0_*) was placed in 20 ml PBS and immersed in an incubator shaker at 37°C. At regular time intervals, three parallel specimens were taken out and pH value was obtained for each specimen (FE 20 pH meter, METTLER TOLEDO, USA). Then the specimens were removed for further analysis. The buffer solution was replaced with an equal volume of fresh PBS.

#### Morphological observation

The dried specimen was adhered by conductive adhesive tape on copper sample stage. Under vacuum the specimen surface was gold-coated for 20 s twice with a sputterer and placed in the Field Emission Scanning Electron Microscope (JSM-6360LV, JEOL Ltd., Japan) for analysis.

### Water uptake and mass loss

At prescribed intervals, the specimens were taken out and rinsed with distilled water for three times. The wet weight (*W*_wet_) was got by removing of surface water and weighing. After dried in vacuum at room temperature until constant weight, measure the weight of samples and recorded as *W*_dry_. The water uptake (%) and weight loss (%) were calculated from the formulation:

(1) $$\text{Water}\;\text{uptake}\;\left(\%\right)=\left(\frac{W_{\text{wet}}{-W}_{\text{dry}}}{W_{\text{dry}}}\right)\times 100\%\;$$

(2) $$\text{Mass}\;\text{loss}\;\left(\%\right)=\left(\frac{W_{0}-W_{\text{dry}}}{W_{0}}\right)\times 100\%$$

At least triplicate parallel replicates were carried out and the average value was calculated for each specimen.

### Molecular weight and distribution

The molecular weight was measured using gel permeation chromatography (Waters 1515, Waters, USA) at 30 °C at a flow rate of 1.0 ml min^−1^ with tetrahydrofuran as the eluent. The molecular weight distribution should be determined relative to a calibration curve generated from polystyrene standards.

### Thermal properties

Differential scanning calorimetry (DSC) was performed on a dynamic mechanical/differential thermal analysis combination Instrument (DSC 2910, TA Instrument, USA). The same thermal history was applied to all the specimens by first heating up to 200 °C and cooling to − 50 °C at 10 °C min^−1^, followed by a second heating run from 50 to 220 °C at 10 °C min^−1^. The glass transition (*T*_g_) was taken at the midpoint of the transition zone on the second heating scan.

### The composition of molecular chain

The transformation of composition of P(TMC-co-DLLA) chain was calculated from *Fox equation*, as shown in Equation 3.

(3) $$T_{g}={\;T}_{\text{gPTMC}}{\;X}_{\text{TMC}}+{\;T}_{\text{gPDLLA}}{\;X}_{\text{DLLA}}$$

Where *T_g_*, *T_g_*_PTMC_ (= −20 °C), and *T_g_*_PDLLA_ (= 57 °C) was glass transition temperature of copolymer, PTMC and PDLLA.

### Mechanical properties

The dried specimen (80 mm length × 10 mm width × 4 mm thickness) was used to three point bending and tensile test at a strain rate of 5 mm min^−1^. Five measurements were carried out at prescribed time point.

### *In vivo* implantation and histological evaluation

*In vivo* biodegradation and biocompatibility of the copolymer strips were evaluated by subcutaneous implantation. All procedures were performed in accordance with the Institutional Animal Care and Use Committee. Pre-weighted specimens with the initial size of 2 × 2 × 4 mm were prepared as described above. The strips were sterilized in 80% ethanol for 30 min and dried in vacuum overnight prior to implantation.

Surgical procedures were carried out under aseptic conditions and materials needed were sterilized before. 12 *Wistar* rats (120–160 g) were divided into four groups (*n* = 3) according to different time points: 1, 2, 4, 8 weeks. Anesthesia was performed by an intraperitoneal injection of 10 wt % chloral hydrate solution (5 ml kg^−1^). After shaving and disinfection, a 10-mm longitudinal incision was made at the dorsocentral region, and the subcutaneous tissue was bluntly dissected. Two specimens were implanted in left and right sides. Then the wound was closed in layers with sutures. Subsequently, the experimental rats were fed in a sterile environment.

At 1, 2, 4 and 8 weeks after implantation, three rats were sacrificed and the six specimens were harvested respectively. Four samples chosen randomly were freed from the surrounding soft tissue to obtain the original implant materials for further biodegradation evaluation. Water uptake, mass loss, change of molecular weight (by GPC), were studied as the same methods abovementioned. Fourier transform infrared analysis (FTIR, Nicolet 6700, Thermo, USA) was conducted to inspect the variation of compositions. The spectral range is 7800–350 cm^−1^, and the resolution ratio is 0.09 cm^−1^. The remanent two were fixed in 4% formaldehyde solution for 24 h, then dehydrated with graded series of ethanol and embedded in paraffin. Thin slices were fabricated and stained with haematoxylin–eosin (H–E) for histological examination.

### Statistical analysis

Results were expressed as mean standard ± deviation. Data analysis was carried out using one-way analysis of variance (ANOVA). Carry out Least Significant Difference (LSD) to show a significant difference between the groups. Differences were considered to be statistical significant at *P* < 0.05.

## Results and discussion

### *In vitro* degradation

#### Morphology variation

**Figure 1:**
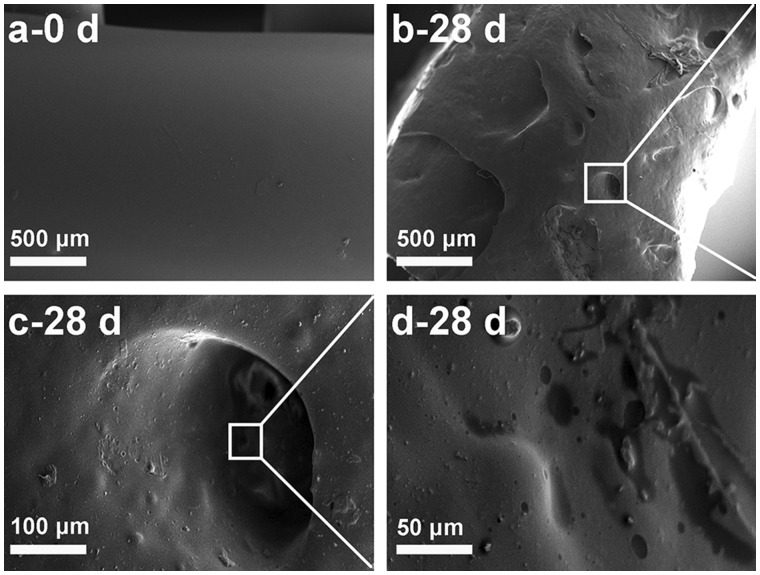
SEM Photographs of P(TMC-co-DLLA) specimen: (**a**) before placed in PBS, (**b**–**d**) superficial morphology after immersing in PBS for 28 days (c and d are magnifying specified locations)

Figure 1 shows the morphological variation of P(TMC-co-DLLA) material before and after immersing in PBS for 28 days. Figure 1c and d are respectively amplified visions of designated sites. Obvious erosion could be found compared with the original smooth surface in Fig. 1a. As displayed in Fig. 1b, the flat surface became uneven, distributing with amounts of holes and sags. Cavities were observed under higher magnification in Fig. 1c, with irregularly hollows appeared on the walls of the cavities as indicated in Fig. 1d. The superficial morphological difference was mainly attributed to the surface erosion of PTMC [18, 19].

#### Mass loss and molecular weight profile

Water uptake plays an important role during degradation of polymers caused by hydrolysis [25]. In general, the lower water uptake capacity is, the slower hydrolysis rate is acquired. Moreover, water uptake depends on the composition of copolymer. It is well known that water is apt to permeate amorphous regions rather than crystalline regions.

**Figure 2:**
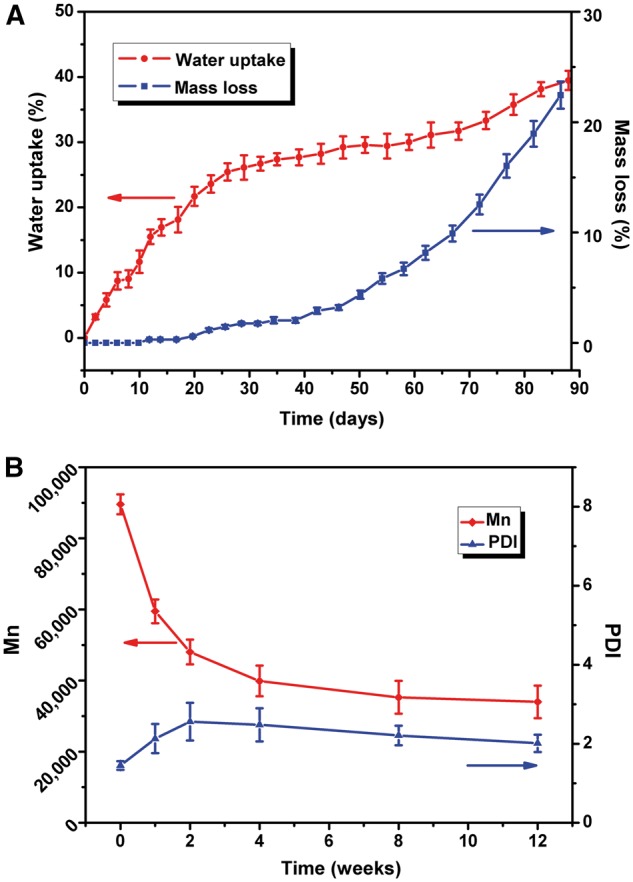
(**A**) Water uptake and mass loss of P(TMC-co-DLLA) in PBS during 90 days. (**B**) M_n_ and PDI in PBS during 12 weeks

Figure 2A shows the mass loss and water uptake profile during 90 days in PBS of pH = 7.4 at 37 °C. Since the prepared P(TMC-co-DLLA) had incompact amorphous structure which made for the penetration of water easily, the water uptake increased steadily during the first 4 weeks, and apparent swelling was observed in this period. After that, the growth of water uptake became slowly until 70 days. Then the tendency speeded up slightly, and cavities appeared owing to the internal collapse.

The mass loss occurred in a lagging tendency. At the initial stage of immersion, the mass loss changed barely. 1 week later, gradually changes were seen in the mass loss. After 30 days the mass loss was about 2%, and reached 6.7% after 60 days, and up to 22.4% after 88 days. Holes and scallops existed on the surface of the sample. After 3 months, the material became brittle and sticky. It was rational that higher water uptake was obtained at later stage for more hydrophilic end groups were exposed along with the disentanglement of macromolecular chains.

**Figure 3:**
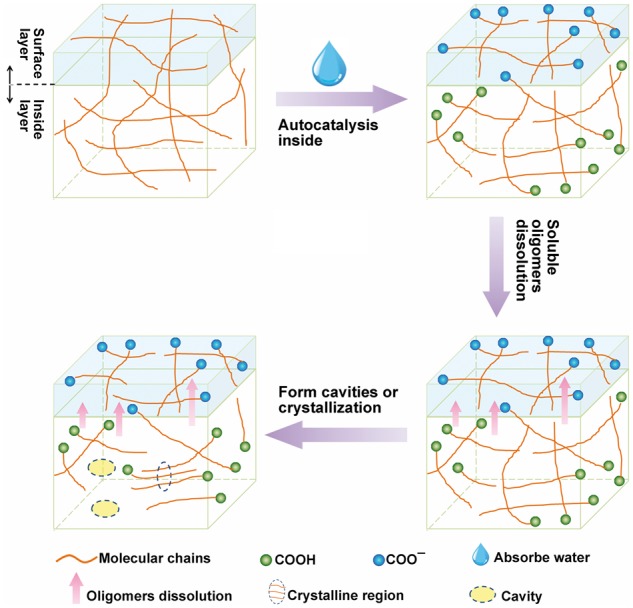
Schematic procedure of the autocatalysis effect and proposed degradation mechanism

The information of molecular weight and polydispersity index changes as a function of degradation time in PBS were obtained via GPC monitoring, which is presented in Fig. 2B. The number average molecular weight (*M_n_*) reduced significantly from initial 89567–59457 after 1 week, and dropped to 48031 after 2 weeks. After immersed for 4 weeks, *M_n_* decreased to 39862, less than 45% of the original value. Nevertheless, the decline rate of *M_n_* turned gentle hereafter, *M_n_* reduced to 35 276 (39% of the original value) and 34 010 (38% of the original value) after 8 weeks and 12 weeks, respectively. Correspondingly, the polydispersity index (PDI) increased steeply in first 2 weeks from initial 1.45–2.56, then underwent a slowly decrease to 2.21 after 8 weeks and to 2.01 after 12 weeks. As depicted in Fig. 3, at the initial stage of degradation, water permeated into amorphous region of the copolymer chains, causing broken of ester bonds and carbonate bonds and generating hydroxyl and carboxyl end groups, whose autocatalytic effect could accelerate scission and disentanglement of polymer chain. Owing to the increasing oligomers, the PDI enhanced following with the decreasing of *M_n_*. However, the automatic acceleration response could not occur on the surface for the neutralization of terminal carboxyl groups by buffer solution. Thus the disentangling was not uniform, internal degradation was faster than exterior decomposition. In the later stage, with dissolving of oligomers, PDI decreased again, which was in agreement with previous studying [25].

#### Thermal and mechanical properties

**Figure 4:**
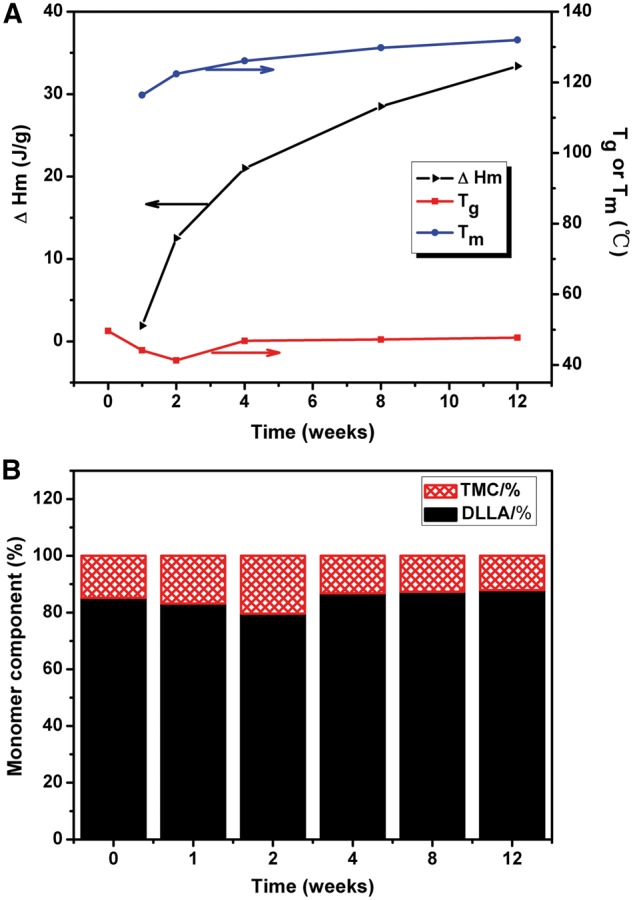
(**A**) *T*_g_, *T*_m_ and △*H*_m_ from DSC curve. (**B**) The monomer components of P(TMC-co-DLLA) in PBS during 12 weeks

Changes of molecular weight influenced the thermal properties of P(TMC-co-DLLA). Glass-transition temperature (*T_g_*), melt temperature (*T*_m_) and melting enthalpy (*△H*_m_) calculated from DSC curve of P(TMC-co-DLLA) in PBS during 12 weeks are shown in Fig. 4A. In the initial period, *T_g_* decreased from initial 49.6 °C to 48.1 °C after 1 week, and to 45.3 °C after 2 weeks. The disassemble oligomers acted as internal plasticizers, resulting in decline of *T_g_*. Similarly, this was accordant with that of the PDI. From 2 to 12 weeks, *T_g_* increased to 51.7 °C. During this period, the decrease of PDI meant low molecular weight oligomers were released outside; as a result, plasticizing effect became diminished.

Noticeably, initial P(TMC-co-DLLA) copolymer didn’t have a *T_m_*, but 1 week later after degradation, DSC curve displayed a weak *T_m_* at 116.4 °C. After 4 weeks, 8 weeks, and 12 weeks, more obvious *T_m_* was revealed at 122.4 °C, 116.2 °C and 129.8 °C. From 1 week to 12 weeks, *ΔH_m_* rose significantly from 1.9 J g^−1^ to 28.5 J g^−1^. It indicated increased regularity of polymer segments along with degradation. This could be attributed to the rapid rupture of amorphous region of PDLLA segments, which promoted additional crystalline regions formation and appearing of *T_m_*, as shown in Fig. 3. The nonuniform molecular weight distribution resulted in a broad melting peak.

Besides, the transformation of composition of P(TMC-co-DLLA) chain was calculated from *Fox equation*, as shown in Equation 3. As exhibited in Fig. 4B, during first 2 weeks, the ratio of TMC in P(TMC-co-DLLA) chain increased, so it could be deduced that the initial degradation rate of PDLLA was faster than PTMC. Whereafter the ratio of TMC brought down, owing to surface erosion of PTMC.

**Figure 5:**
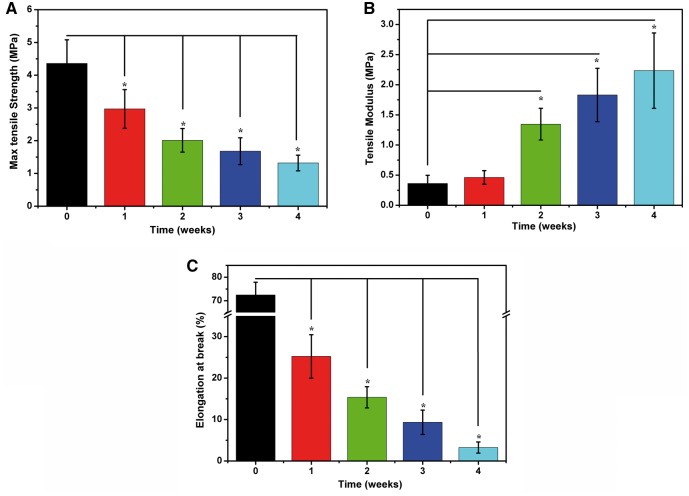
Tensile properties of P(TMC-co-DLLA) in PBS during 4 weeks: (**A**) max tensile strength; (**B**) tensile modulus; (**C**) elongation at break. *P < 0.05 between the marked group with the group before degradation

The degradation of P(TMC-co-DLLA) in PBS solution was a combinational hydrolysis of ester bonds and carbonate ester bonds, including the breakage of backbone, hydrolysis of side chains, and fracture of crosslink bonds in side chains. The degeneration of mechanical strength was related to backbone fracture and decrease in *M_n_* [29]. Figure 5 shows tensile properties changes in PBS during 4 weeks. After 4 weeks the max tensile strength decreased from 4.36 MPa to 1.32 MPa, and elongation at break drastically declined to 3.25%. Moreover the rapid increase of tensile modulus from initial 0.36 MPa to 2.23 MPa after 4 weeks demonstrated that the material became so hard and brittle that elastic deformation was difficult to happen.

### *In vivo* degradation

#### Macroscopic evaluation

**Figure 6:**
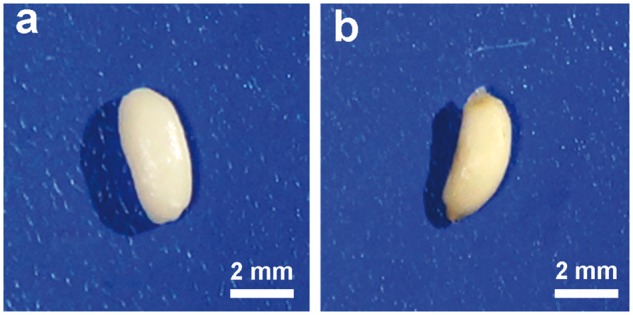
Digital photos of P(TMC-co-DLLA) at different implantation time in subcutaneous region of rats: (**a**) 1 week, (**b**) 4 weeks

Figure 6 shows the general observation of specimen after implantation *in vivo* for 1 week and 4 weeks. After implanting for 1 week, strip still displayed smooth surface, and muscle tissue could be stripped easily. However, after 4 weeks, strips became sticky and a little rough, which implied degradation occurred. Enzymes or other chemical factors were able to cause *in vivo* degradation of polymer materials. Both enzymatic degradation and hydrolytic degradation mechanism can work separately or simultaneously [24].

#### Mass loss and molecular weight

**Figure 7:**
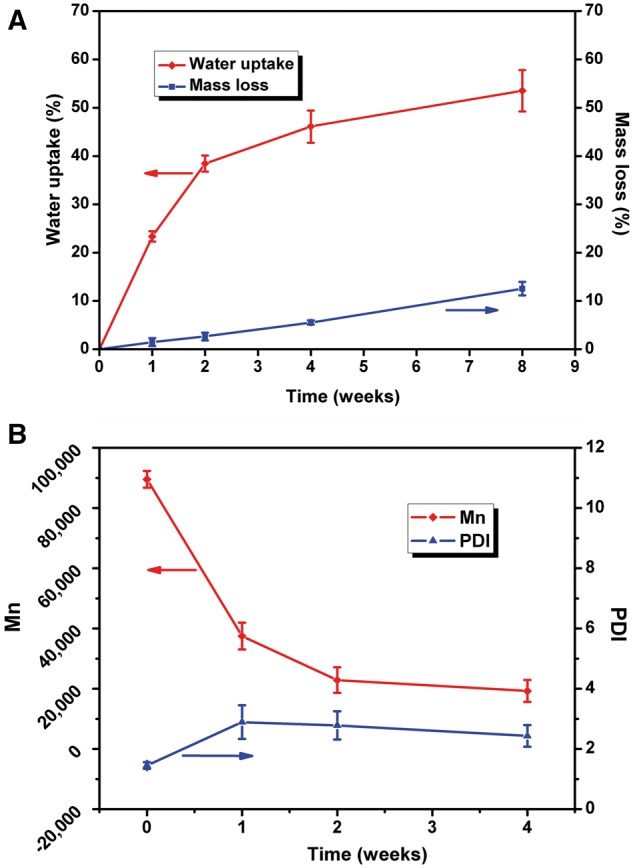
(**A**) Water uptake and mass loss of P(TMC-co-DLLA) during 8 weeks in subcutaneous region of rats. (**B**) M_n_ and PDI during 4 weeks

Figure 7A shows water uptake and mass loss of P(TMC-co-DLLA) during 8 weeks. The P(TMC-co-DLLA) used in this study was amorphous with incompact macromolecular chains; water can easily penetrate molecular chain structure, which resulted in rapid increase of water uptake. Following with scission of polymer chain, the degradation products, such as soluble oligomers were released, so that micropores and voids appeared, as exhibited in Fig. 3. After 4 weeks, materials swelled, and a few holes generated inside the material. Water uptake rate also slowed, as shown in Fig. 7A. Mass loss was an inevitable result of polymer degradation. One week after implantation, the mass loss (%) was up to 1.5%. With the advance of degradation, a growing number of molecular chains broke, at the same time more and more low molecular oligomers were disintegrated and dissolved. 2 weeks later, the mass loss of P(TMC-co-DLLA) reached 2.6%, 4 weeks later reached 5.6%, and 8 weeks later reached 12.6%. Results above all overtop the relevant value at the same observation time obtained in PBS. It can be affirmed the degradation rate *in vivo* was faster than *in vitro*, which may ascribe to enzymatic degradation and cytophagocytosis apart from hydrolysis.

The *in vivo* variation of M_n_ and PDI of P(TMC-co-DLLA) during 4 weeks implantation was consistent with the trend *in vitro*. As indicated in Fig. 7B, the M_n_ of P(TMC-co-DLLA) decreased significantly from initial 89 567–37 490 after 1 week, to 22 864 after 2 weeks, and to 17242 after 4 weeks. Similar to mass loss, the *M_n_ in vivo* also declined faster than that *in vitro*. As demonstrated in Fig. 2B, the M_n_ dropt merely to 39 864 after 4 weeks in PBS. However, the PDI of P(TMC-co-DLLA) showed different trend. It increased from initial 1.45–2.89 after 1 week. Owing to the self-acceleration catalysis and cellular response, more oligomers with different molecular weight were generated and led to augment of PDI 1 week later. After 2 weeks, along with gradually diffused out and dissolved of oligomers, PDI fell back to 2.78, and continued decreasing to 2.43 after 4 weeks.

**Figure 8:**
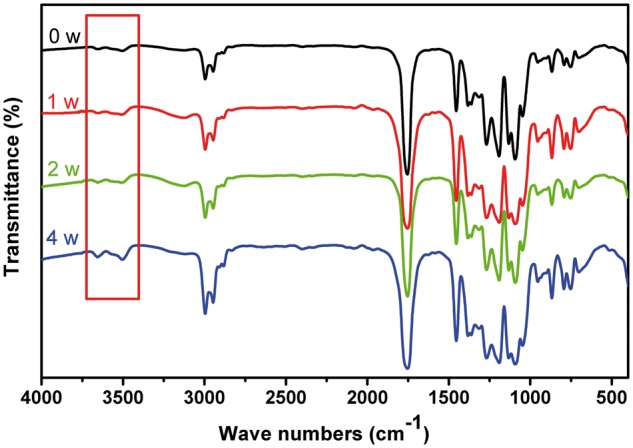
FT-IR Spectra of P(TMC-co-DLLA) during 4 weeks implantation in subcutaneous region of rats

Figure 8 shows the FI-IR spectra of P(TMC-co-DLLA) for *in vivo* implantation for 4 weeks. Compared with the original sample, the stretching bands at 3514 cm^−1^ from O–H groups enhanced. It is rational that the P(TMC-co-DLLA) hydrolytically degraded and regenerated lots of hydroxyl groups.

#### Histological evaluation

**Figure 9:**
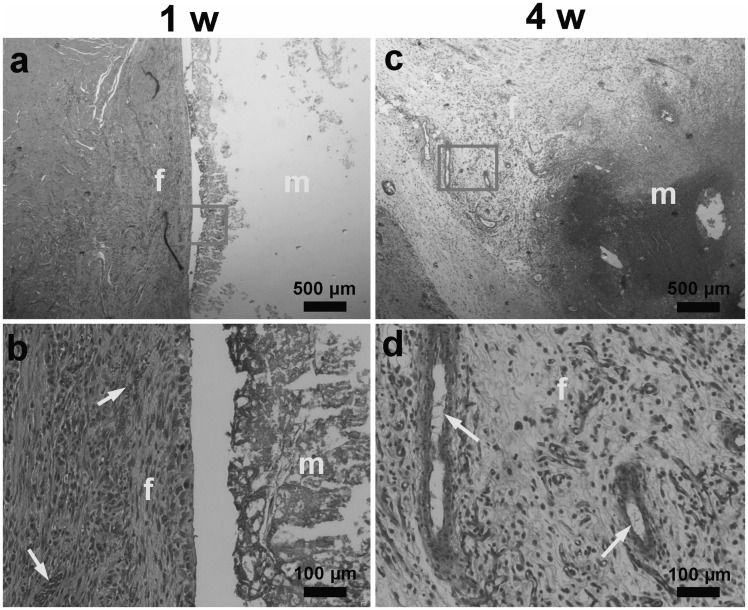
Histological evaluation of the harvested implants, H–E stain: (**a** and **b**) after 1 week; (**c** and **d**) after 4 weeks. m: material area; f: fibrous connective tissue; arrow: blood vessel; circle: magnifying specified locations; broken line: boundary

Figure 9 shows histological evaluation from H-E staining of the harvested implants after 1 week and 4 weeks. In Fig. 9a and 9b, material kept intact, encompassed by fibrous connective tissue with blood capillaries inside, which were advantageous for carting of degraded products, and boundary kept distinct. Figure 9c and 9d showed that after 4 weeks, material area tattered and degraded into scattered parts, surrounded by more fibrous connective tissue than 1 week. Macrophagocytes were observed in-growth, indicating the participating of phagocytosis and degradation of cells. It has been confirmed that reactive oxygen species secreted by phagocytic cells play an important role in the *in vivo* degradation of PTMC [22, 23], so phagocytosis of cells may mainly aim at TMC fragments. Although only copolymer with the ratio of TMC: DLLA = 15: 85 was investigated here, it is logical to speculate that the degradation rate of copolymer can be modulated conveniently by altering the proportion of TMC/DLLA, which led to the different contributions of two kinds of degradation mechanism.

## Conclusions

The degradation behaviors of P(TMC-DDLA) copolymer (15:85) were investigated under *in vitro* and *in vivo* conditions. Changes of surface morphology were in agreement with surface erosion process and bulk degradation. The ratio of TMC in copolymer increased at initial stage, demonstrating that ester bonds from DLLA units were more preferentially broken to hydrolytic cleavage than the carbonate ones. Degradation brought about decline in molecular weight and attenuation of mechanical properties. Furthermore, more rapid degradation rate was observed under *in vivo* condition owing to the participation of phagocytosis. Thus, degradation rate can be regulated through variation of composition according to the different requirements. Therefore, P(TMC-co-DLLA) copolymer is a promising candidate for tissue repairing. 
